# Public Interest in Cognitive Impairment: An Analysis of the Top 50 Articles on Cognitive Impairment on Altmetric

**DOI:** 10.1155/2020/1836471

**Published:** 2020-01-28

**Authors:** Yeo Jin Kim, Yerim Kim, Jee-Eun Kim, Yoo Hwan Kim, Dae Young Yoon, Jong Seok Bae

**Affiliations:** ^1^Department of Neurology, Chuncheon Sacred Heart Hospital, Hallym University College of Medicine, Chuncheon, Republic of Korea; ^2^Department of Neurology, Kangdong Sacred Heart Hospital, Hallym University College of Medicine, Seoul, Republic of Korea; ^3^Department of Neurology, Seoul Medical Center, Seoul, Republic of Korea; ^4^Department of Neurology, Hangang Sacred Heart Hospital, Hallym University College of Medicine, Seoul, Republic of Korea; ^5^Department of Radiology, Kangdong Sacred Heart Hospital, Hallym University College of Medicine, Seoul, Republic of Korea

## Abstract

**Background:**

As the average life expectancy continues to increase, interest in cognitive impairment is increasing. Nowadays, as social media expands its reach, academic research is spreading through social media, changing the way and speed by which research is propagated and also who consumes this content. Therefore, using Altmetric, a new web-based set of metrics that analyzes the impact of content on social media platforms, we investigated the characteristics of influential research articles on the topic of cognitive impairment in social media.

**Methods:**

An Altmetric Explorer search was performed on May 25, 2018, to extract the following information: (i) journal name, (ii) journal impact factor (IF), (iii) year of publication, (iv) article topic, (v) article type, and (vi) cognitive impairment subtype.

**Results:**

The journal “Neurology” was the most cited journal for cognitive impairment articles shared on social media. Among the various types of cognitive impairment, most articles were related to dementia (all subtypes), Alzheimer's disease, and aging. The most common article type was original scientific paper, especially cohort study. The most popular topic was the identification of protective or risk factors for cognitive impairment.

**Conclusion:**

The characteristics of articles with a high Altmetric Attention Score were somewhat different from those of articles with a high number of traditional citations. Social media had the disadvantage that it was difficult to verify the authenticity of the primary source in question, but the advantage was that it could immediately determine the trends regarding how information about that source was being shared and consumed. Therefore, it may be advisable to use Altmetric analysis in combination with traditional methods of evaluating the research articles to understand the dissemination of scientific research and to direct future research.

## 1. Introduction

As social media has evolved, academic research has spread rapidly to the public through this channel. As the life span of mankind has increased, interest in maintaining a healthy later life has increased, with a focus on cognitive function being central to this interest. Social media, therefore, is replete with information about cognitive function. Altmetrics is a new web-based set of metrics that analyzes the impact of social media platforms such as Twitter and Facebook [1]. The Altmetric Attention Score (AAS) is a weighted score, calculated using differential weights for multiple factors depending on the amount of visibility they promote [2]. Research articles with a high AAS meant that they got more attention from social media. Traditionally, citation-based metrics such as impact factor and h-index have been used to determine the impact of articles [3]. Traditional citations take a considerable amount of time to accumulate after publication, whereas AAS is updated as soon as attention occurs online, so AAS could be used as an ancillary tool to evaluate the influence of the research articles, immediately.

Therefore, we would like to investigate which of the cognitive impairment-related studies received high AAS and consider the impact of these trends.

## 2. Methods

### 2.1. Search Engine

Altmetric Explorer (Altmetric, London, UK) is a web-based application that can be used to search the following sources of research output, to yield the most-relevant and up-to-date picture of the following types of online activity and discussion from public policy documents, blogs, mainstream media, citations data, online reference managers, research highlights, postpublication peer-review platforms, social networks, Wikipedia, Open Syllabus Project, multimedia and other online platforms, and patents [4].

We followed the methods of Kim et al. [5]. The AAS and the Altmetric donut are designed to make it easy to identify how much and what type of attention a particular research output has received [6]. The AAS is automatically calculated using an automated algorithm created by the company Altmetric, based on the weighted count of all the attention a research output has received. It is based on three main factors: volume, sources, and authors. Each color of the Altmetric donut represents a different source of attention [6].

### 2.2. Search Strategy

An Altmetric Explorer search was performed on May 25, 2018, for articles published in the 91 journals included in the 2015 InCites™ Journal Citation Report® grouped under the subject categories of clinical neurology, neuroimaging, neurosciences, radiology, nuclear medicine and medical imaging, surgery, and general and internal medicine.

Two researchers (Y. J. Kim and Y. Kim) read the full papers of the top 50 articles and extracted the following information by consensus: (i) journal name, (ii) journal impact factor (IF), (iii) year of publication, (iv) article topic, (v) article type, and (vi) cognitive impairment subtype.

To clarify the AAS for the cognitive impairment field, we excluded articles that suggested composite outcomes including psychiatric disorder. If the outcomes were treated separately, the article was included in this study.

## 3. Results

Altmetric Explorer identified 43,410 articles as being referred to online. The AAS, journal name, impact factor (IF) ranking, publication year, and number of citations of the top 50 articles are summarized in Table 1. Because Altmetrics started collecting data only during the second half of 2011, the articles before 2010 were rarely listed. The number of papers on this list peaked in 2017 (*n* = 20). Eight articles originated from the journal *Neurology* followed by JAMA Internal Medicine and JAMA, each with five articles. All but one of the articles were published in journals in the highest IF quartile (Table 2). Regarding cognitive impairment subtypes, 20 articles were on dementia, 14 were on Alzheimer's disease, 10 were on aging, and 2 were on chronic traumatic encephalopathy (Table 3). Regarding article types, 40 articles were original scientific papers, 4 were related to systematic reviews and meta-analyses, 2 were related to guidelines and advisory documents, 3 were related to reviews, and 1 was related to an editorial (Table 4). The most common topic was risk and protective factors for cognitive impairment (*n* = 27) (Table 5). In particular, as reports of cohort studies and editorials were both popular in social media, people were interested in the result that sugar-sweetened beverages increased the incidence of dementia (#3 and #41 in Table 1) [7, 8]. However, green leafy vegetables and a Mediterranean diet had a protective effect against cognitive decline (#5 and #9) [9, 10]. MIND diet was also associated with decreased risk of Alzheimer's disease [11]. Glucose and vitamin D levels were associated with the risk of cognitive decline (#17 and #18) [12, 13]. Alcohol intake and marijuana use were associated with cognitive health. Marijuana decreased perfusion of hippocampus [14]. However, there were discrepancies in opinion on alcohol consumption. In a cohort study published in 2014, excessive alcohol consumption of more than the average daily amount of 36 g in midlife males was associated with faster cognitive decline (#28) [15], whereas in a study published in 2017, it was found that regular, moderate alcohol intake enhanced cognitively healthy longevity (#21) [16]. Leisure activity and midlife cardiovascular fitness were also associated with the risk of dementia. Some studies reported that comorbid conditions such as hearing loss (#19) [17], poor sleep (#26) [18], and chronic pain (#46) [19] deteriorated cognitive health. Environmental factors such as living near a major road (#4) [20] and lead exposure (#32) [21] affected cognitive health. Furthermore, medications such as proton pump inhibitors and anticholinergics were related to cognitive function. Over-the-counter supplement use was also effective against cognitive decline (#35) [22]. Bilingualism (#12, #44, and #47) [23–25] and marriage type (#50) [26] could also affect cognitive health.

## 4. Discussion

Here, we summarized the top 50 articles using AAS. The mean AAS was 1171.6, and the mean of the number of citations was 109.5. Articles were published the most in 2017, and the journal “Neurology” was the most popular originated journal. Among the types of cognitive impairments, most articles were related to dementia, Alzheimer's disease, and aging. The most common article type was original scientific paper, especially cohort study. The most popular topic was the protective or risk factor.

Most of the articles were published in 2017, likely because we performed the Altmetric Explorer search on May 25, 2018. The same method used in the article written by Kim et al., co-author of this article, was used here [5]. AAS was sensitive to the latest content, and recent publications tended to receive higher AAS. For example, the Altmetric top 20 list of Parkinson's disease research published in 2017 did not include any papers that were reported to be the most influential in 2011. Most research articles included in the list were 2016 or 2015 studies [27]. However, in our report, two articles, about leisure activities and bilingualism as protective factors, were included in the top 50 list even though they were published before 2011. Therefore, it was suggested that the influence of important articles through social media might be weakened, but it did not disappear even after some time.

The Altmetric score generally reflects the interest of the lay public, not the scholar, because it measures all social media sources. In the past, academic research articles were typically the exclusive domain of scholars, but nowadays, because of the development of media, research articles are consumed by the general public as well as scholars. So far, the research article was a means of communication for researchers and the degree of citation was an important indicator for evaluating the quality of the articles. However, due to the development of social media, expertise on topics such as medicine and disease was widely publicly available and research that received public attention could be more readily supported. Nonetheless, since public and professional interests differ, citation index and social media indicators were not always in agreement. This analysis showed that social media audiences were most interested in modifiable risk factors related to cognitive impairment. Diet and nutrients, social factors, alcohol and drugs, and medications investigated in articles with high AAS were risk factors that could be changed through behavioral modification. In addition, people were also interested in treatment and prevention, and in particular, they were more concerned with nonpharmacologic management than pharmacologic management among treatment. In the absence of clear pharmacological treatment for dementia, it seemed that there was a relatively greater interest in factors that could help improve cognitive function, as well as prevention and nonpharmacological treatment. The top-ranked article also reported about chronic traumatic encephalopathy, which could be avoided if exposure to trauma was minimized.

Disease groups with higher AAS were all subtypes of dementia, Alzheimer's disease, and aging. It was also worth noting that interest in aging without dementia was high. This could be related to the higher interest in prevention than treatment. As there was no disease-modifying treatment for dementia, it seemed that there was more interest in how to maintain cognitive function in healthy elderly individuals. Indeed, even among scholars, the development of preventive methods to reduce cognitive impairment through lifestyle changes has been emphasized over treatment. The paper by Kivipelto and collaborators was a randomized controlled study investigating whether an intervention of diet, exercise, cognitive training, and vascular risk monitoring could prevent cognitive decline of normal elderly individuals. This paper, published in 2012 in The Lancet, was one of the most cited in the Altmetric top 50 article list for cognitive impairment (939 AAS, 604 citations) [28].

On the other hand, there was less interest in professional contents such as pathomechanism or biomarkers of dementia. For example, in Alzheimer's disease, amyloid and tau proteins are related to the pathomechanisms of the disease. As a result, the technology of detecting amyloid and tau protein in CSF or visualization of amyloid and tau using brain imaging has been highly studied [29]. However, none of these studies were included in the Altmetric top 50 list. These studies were not less important because of the low AAS of pathomechanisms or biomarker-related studies. Since the research that interests the public was not necessarily the most relevant and the Altmetric score based on social media is a reflection of the public's interest, it is difficult for AAS to accurately reflect the importance or quality of research. In addition, since social media lacks the ability to verify whether the information was real or fake [30], the possibility of misleading research spreading through social media could lead to a high AAS, despite lack of credibility or relevance. Therefore, it could be dangerous to evaluate the value of academic publications by AAS alone.

Nevertheless, the world is rapidly evolving to use the web to share information rather than more traditional media, and because many journals are converting to open access, the information inherent in social media and other web platforms cannot be ignored. As an additional tool for evaluating a paper or topic, using AAS in combination with traditional methods can help better understand the impact of scientific findings.

## 5. Conclusions

We reviewed popular articles on cognitive impairment using Altmetric analysis. In the analysis, the most salient characteristics of top articles of interest were those that were most recently published, cohort studies, and those published in the journal Neurology. The protective or risk factors associated with cognitive impairment was the topic of greatest interest. In order to understand the flow of scientific research, we suggest using Altmetric analysis as an alternative tool, along with traditional tools for evaluating article impact.

## Figures and Tables

**Table 1:** Top 50 articles with the highest Altmetric Attention Scores (AASs).

Rank	Article title	AAS	Journal name	Impact factor ranking	Date of publication (YYMMDD)	Number of citations
1	Clinicopathological evaluation of chronic traumatic encephalopathy in players of American football	3674	JAMA: Journal of the American Medical Association	Q1	2017-07-25	170

2	Dementia prevention, intervention, and care	3294	The Lancet	Q1	2017-07-19	466

3	Sugar- and artificially sweetened beverages and the risks of incident stroke and dementia	3215	Stroke	Q1	2017-01-01	27

4	Living near major roads and the incidence of dementia, Parkinson's disease, and multiple sclerosis: a population-based cohort study	2924	The Lancet	Q1	2017-01-04	114

5	Nutrients and bioactives in green leafy vegetables and cognitive decline: prospective study	2538	Neurology	Q1	2017-12-20	15

6	A comparison of the prevalence of dementia in the United States in 2000 and 2012	1610	JAMA Internal Medicine	Q1	2016-11-21	158

7	Incidence of dementia over three decades in the framingham heart study	1544	New England Journal of Medicine	Q1	2016-02-11	277

8	Mixed pathologies including chronic traumatic encephalopathy account for dementia in retired association Football (soccer) players	1532	Acta Neuropathologica	Q1	2017-02-15	32

9	Mediterranean diet and age-related cognitive decline: a randomized clinical trial	1486	JAMA Internal Medicine	Q1	2015-05-11	181

10	Association of proton pump inhibitors with risk of dementia: a pharmacoepidemiological claims data analysis	1458	JAMA Neurology	Q1	2016-02-15	191

11	Alzheimer's disease drug-development pipeline: few candidates, frequent failures	1281	Alzheimer's Research & Therapy	Q1	2014-07-03	509

12	Bilingualism delays age at onset of dementia, independent of education and immigration status	1209	Neurology	Q1	2013-01-01	146

13	Circadian rest-activity pattern changes in aging and preclinical alzheimer disease	1183	JAMA Neurology	Q1	2018-01-29	19

14	Evidence of amyloid-beta cerebral amyloid angiopathy transmission through neurosurgery	1097	Acta Neuropathologica	Q1	2018-02-15	12

15	MIND diet associated with reduced incidence of Alzheimer's disease	1074	Alzheimer's & Dementia: the Journal of the Alzheimer's Association	Q1	2015-02-11	99

16	Practice guideline update summary: mild cognitive impairment: report of the guideline development, dissemination, and implementation subcommittee of the american academy of neurology	1038	Neurology	Q1	2017-12-27	86

17	Glucose levels and risk of dementia	1020	New England Journal of Medicine	Q1	2013-08-08	292

18	Vitamin D and the risk of dementia and alzheimer disease	989	Neurology	Q1	2014-08-05	151

19	Hearing loss and cognitive decline in older adults	956	JAMA Internal Medicine	Q1	2013-02-25	397

20	Midlife cardiovascular fitness and dementia: a 44-year longitudinal population study in women	954	Neurology	Q1	2018-03-14	12

21	Alcohol intake and cognitively healthy longevity in community-dwelling adults: the rancho bernardo study	950	Journal of Alzheimer's Disease	Q2	2017-06-28	2

22	Cumulative use of strong anticholinergics and incident dementia: a prospective cohort study	942	JAMA Internal Medicine	Q1	2015-01-26	251

23	A 2 year multidomain intervention of diet, exercise, cognitive training, and vascular risk monitoring versus control to prevent cognitive decline in at-risk elderly people (FINGER): a randomised controlled trial	939	The Lancet	Q1	2015-03-11	604

24	Impact of person-centred care training and person-centred activities on quality of life, agitation, and antipsychotic use in people with dementia living in nursing homes: a cluster-randomised controlled trial	931	PLoS Medicine	Q1	2018-02-06	13

25	Leisure activities and the risk of dementia in the elderly	931	New England Journal of Medicine	Q1	2003-06-19	868

26	Poor sleep is associated with CSF biomarkers of amyloid pathology in cognitively normal adults	930	Neurology	Q1	2017-07-07	25

27	A qualitative impairment in face perception in Alzheimer's disease: evidence from a reduced face inversion effect	903	Journal of Alzheimer's Disease	Q2	2016-02-26	5

28	Alcohol consumption and cognitive decline in early old age	895	Neurology	Q1	2014-01-28	46

29	Aerobic exercise and vascular cognitive impairment	884	Neurology	Q1	2016-10-19	25

29	Risk of pneumonia associated with incident benzodiazepine use among community-dwelling adults with alzheimer disease	884	Canadian Medical Association Journal	Q1	2017-04-10	16

31	Summary of the evidence on modifiable risk factors for cognitive decline and dementia: a population-based perspective	845	Alzheimer's & Dementia: The Journal of the Alzheimer's Association	Q1	2015-06-06	248

32	Association of childhood blood lead levels with cognitive function and socioeconomic status at age 38 years and with IQ change and socioeconomic mobility between childhood and adulthood	835	JAMA: Journal of the American Medical Association	Q1	2017-03-28	51

33	Slow wave sleep disruption increases cerebrospinal fluid amyloid-beta levels	832	Brain: A Journal of Neurology	Q1	2017-08-01	53

34	Discriminative properties of hippocampal hypoperfusion in marijuana users compared to healthy controls: implications for marijuana administration in Alzheimer's dementia	805	Journal of Alzheimer's Disease	Q2	2016-11-24	3

35	Over-the-counter supplement interventions to prevent cognitive decline, mild cognitive impairment, and clinical alzheimer-type dementia: a systematic review	800	Annals of Internal Medicine	Q1	2017-12-19	12

36	Effect of omega-3 fatty acids, lutein/Zeaxanthin, or other nutrient supplementation on cognitive function: The AREDS2 randomized clinical trial	794	JAMA: Journal of the American Medical Association	Q1	2015-08-25	56

37	Effect of vitamin E and memantine on functional decline in alzheimer disease: the TEAM-AD VA cooperative randomized trial	788	JAMA: Journal of the American Medical Association	Q1	2014-01-01	197

38	Does cognitive training prevent cognitive decline?: a systematic review	787	Annals of Internal Medicine	Q1	2017-12-19	14

39	Physical exercise moderates the relationship of apolipoprotein E (APOE) genotype and dementia risk: a population-based study	784	Journal of Alzheimer's Disease	Q2	2016-01-01	7

40	Physical activity interventions in preventing cognitive decline and alzheimer-type dementia: a systematic review	762	Annals of Internal Medicine	Q1	2017-12-19	23

41	Sugar-sweetened and artificially sweetened beverages in relation to stroke and dementia	753	Stroke	Q1	2017-01-01	2

42	APOE DNA methylation is altered in lewy body dementia	743	Alzheimer's & Dementia: The Journal of the Alzheimer's Association	Q1	2018-03-12	2

43	Evaluation of the safety, tolerability, and efficacy of pimavanserin versus placebo in patients with Alzheimer's disease psychosis: a phase 2, randomised, placebo-controlled, double-blind study	742	Lancet Neurology	Q1	2018-03-01	18

44	Does bilingualism influence cognitive aging?	736	Annals of Neurology	Q1	2014-06-02	102

45	Blood-brain barrier leakage in patients with early alzheimer disease	735	Radiology	Q1	2016-05-31	93

46	Association between persistent pain and memory decline and dementia in a longitudinal cohort of elders	723	JAMA Internal Medicine	Q1	2017-06-05	15

47	Bilingualism: consequences for mind and brain	719	Trends in Cognitive Sciences	Q1	2009-01-01	328

48	Sugary beverage intake and preclinical Alzheimer's disease in the community	712	Alzheimer's & Dementia: The Journal of the Alzheimer's Association	Q1	2017-03-05	5

48	Testosterone treatment and cognitive function in older men with low testosterone and age-associated memory impairment	712	JAMA: Journal of the American Medical Association	Q1	2017-02-21	53

50	Marriage and risk of dementia: systematic review and meta-analysis of observational studies	708	Journal of Neurology, Neurosurgery & Psychiatry	Q1	2017-10-31	9

**Table 2:** Journals with top 50 articles, ranked according to the AAS.

Rank	Journal name	Impact factor ranking	Number of articles
1	Neurology	Q1	8
2	JAMA Internal Medicine	Q1	5
2	JAMA: Journal of the American Medical Association	Q1	5
4	Alzheimer's & Dementia: The Journal of the Alzheimer's Association	Q1	4
4	Journal of Alzheimer's Disease	Q2	4
6	Annals of Internal Medicine	Q1	3
6	New England Journal of Medicine	Q1	3
6	The Lancet	Q1	3
9	Acta Neuropathologica	Q1	2
9	JAMA Neurology	Q1	2
9	Stroke	Q1	2
12	Alzheimer's Research & Therapy	Q1	1
12	Annals of Neurology	Q1	1
12	Brain: A Journal of Neurology	Q1	1
12	Canadian Medical Association Journal	Q1	1
12	Journal of Neurology, Neurosurgery & Psychiatry	Q1	1
12	Lancet Neurology	Q1	1
12	PLoS Medicine	Q1	1
12	Radiology	Q1	1
12	Trends in Cognitive Sciences	Q1	1

**Table 3:** Number of articles with top 50 AASs according to cognitive impairment subgroups.

Subgroup	Number of articles
All dementia	20
Alzheimer's disease	14
Aging	10
Chronic traumatic encephalopathy	2
Mild cognitive impairment	1
Vascular cognitive impairment	1
Cerebral amyloid angiopathy	1
Lewy body dementia	1

**Table 4:** Number of articles with top 50 AASs according to article types.

Subgroup	Number of articles
Original scientific papers	40
Randomized clinical trial	8
Cohort study	22
Cross-sectional study	3
Case-control study	4
Case series	3
Systematic reviews and meta-analyses	4
Guidelines and advisory documents	2
Reviews	3
Editorial	1

**Table 5:** Number of articles with top 50 AASs according to subject categories.

Subject category	Number of articles
Risk factors and protective factors	27
Diet and nutrients	9
Social factor	4
Alcohol and drugs	3
Associated disease	3
Medication	2
Pollution	2
Sleep pattern	2
Global modifiable risk factors	1
Leisure activity	1
Prevention and treatment	13
Pathomechanism and brain biopsy	5
Epidemiology	2
Biomarker	1
Disease characteristics	1
Complications	1

## Data Availability

The data used to support the findings of this study were supplied by “Advanced Search” in Altmetric Explorer (https://www.altmetric.com/explorer, Altmetric LLP, London, UK).
